# Two New Diterpenoids from *Biscogniauxia sp*. and Their Activities

**DOI:** 10.3389/fchem.2021.749272

**Published:** 2021-09-01

**Authors:** Huan Zhao, Yue Liu, Meng Zhang, Guo-dong Chen, Dan Hu, Liang-dong Guo, Zhong-xiang Zhao, Hui Zhi, Hao Gao

**Affiliations:** ^1^ College of Traditional Chinese Medicine, Jinan University, Guangzhou, China; ^2^ Institute of Traditional Chinese Medicine and Natural Products, College of Pharmacy, Jinan University, Guangzhou, China; ^3^ State Key Laboratory of Mycology, Institute of Microbiology, Chinese Academy of Sciences, Beijing, China; ^4^ School of Chinese Materia Medica, Guangzhou University of Chinese Medicine, Guangzhou, China

**Keywords:** *Biscogniauxia sp*, diterpenoids, isopimarane type, abietane type, Alzheimer’s disease

## Abstract

Two new diterpenoids, including a *seco*-isopimarane type (1) and an abietane type (2), were isolated from *Biscogniauxia* sp. (71-10-1-1). Their structures, including absolute configurations, were elucidated by NMR spectroscopic analyses, X-ray crystallography, ^13^C chemical shifts calculations, and ECD calculations. This is the first report of diterpenoids from *Biscogniauxia* sp. Furthermore, short-term memory enhancement against Alzheimer’s disease (AD), anti-inflammatory, and cytotoxic activities of 1–2 were also evaluated. The results showed that compound 1 exhibited short-term memory enhancement activity against AD.

## Introduction

*Biscogniauxia sp*. belongs to the Xylariaceae family, growing on the bark of trees and shrubs, preferably on dead or dying branches. Since the first report on the secondary metabolites in 2005 (Evidente et al., 2005), nearly 50 secondary metabolites have been reported, including azaphilones (Cheng et al., 2012), meroterpenoids (Zhao et al., 2017; Zhao et al., 2019; Zhao et al., 2021), sesquiterpenes (Amand et al., 2012), aromatics (Evidente et al., 2005; Cheng et al., 2012; Cheng et al., 2011), isocoumarins (Evidente et al., 2005; Cheng et al., 2012; Amand et al., 2012; Sritharan et al., 2019), amides (Cheng et al., 2012; Cheng et al., 2011), and cyclopeptides (Wu et al., 2016), which exhibit a widespread of bioactivities, including antimicrobial (Cheng et al., 2012), enzyme inhibitory (Wu et al., 2016), inhibitory of seed germination (Evidente et al., 2005), anti-inflammatory (Zhao et al., 2021), or short-term memory enhancement in AD flies activities (Zhao et al., 2017).

**Figure:**
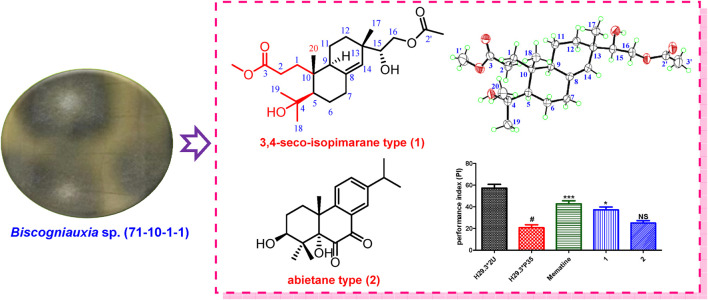
GRAPHICAL ABSTRACT

**FIGURE 1:**
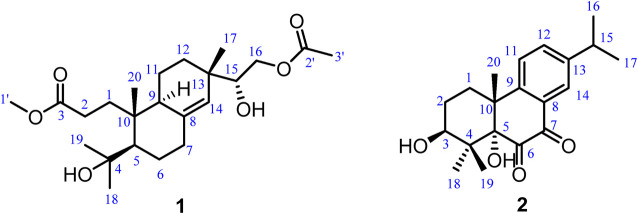
Chemical structures of 1–2.

In our previous chemical investigation of a fungal strain of *Biscogniauxia* sp. (No.71-10-1-1), structurally diverse active meroterpenoids (diisoprenyl-cyclohexene/ane-type) and their dimers with new skeleton were identified (Zhao et al., 2017; Zhao et al., 2019; Zhao et al., 2021), which showed that this strain can produce natural products with rich chemical diversity. Further chemical investigation on this fungal strain was carried out, which led to the isolation of two new diterpenoids, including a *seco*-isopimarane type (1) and an abietane type (2). Their structures, including absolute configurations, were elucidated by NMR spectroscopic analyses, X-ray crystallography, ^13^C chemical shifts calculations, and ECD calculations. Details of the structure elucidations for 1–2 are reported herein (Figure 1). In addition, short-term memory enhancement against AD, anti-inflammatory, and cytotoxic activities of 1–2 were also evaluated. The results showed that 1 exhibited short-term memory enhancement activity.

## Results and Discussion

**FIGURE 2:**
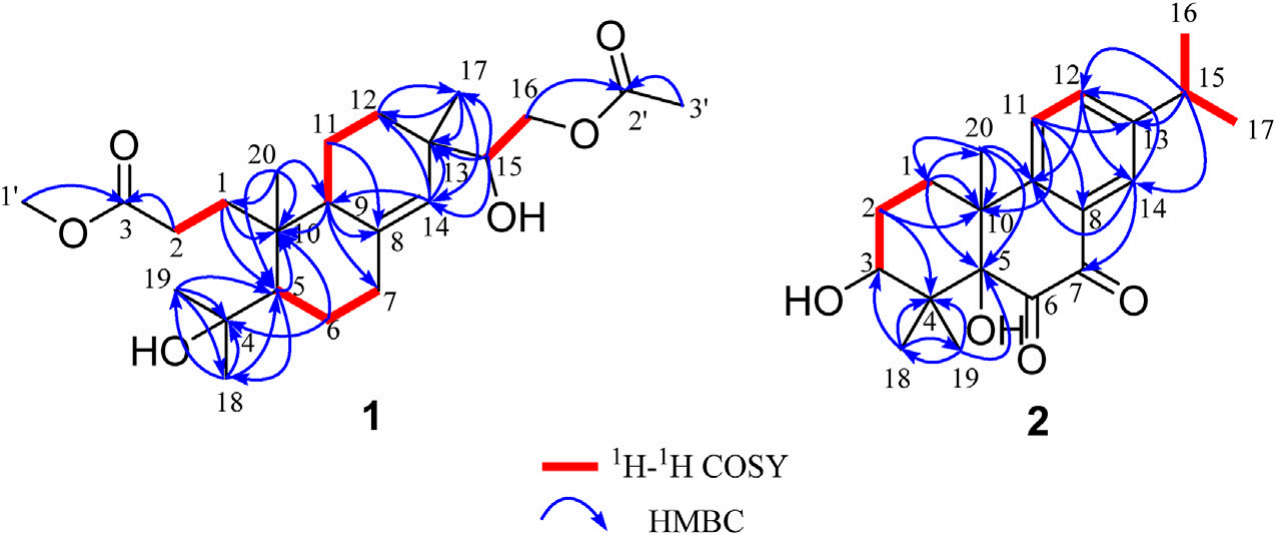
Key ^1^H-^1^H COSY and HMBC correlations and the planar structures of 1–2.

**FIGURE 3:**
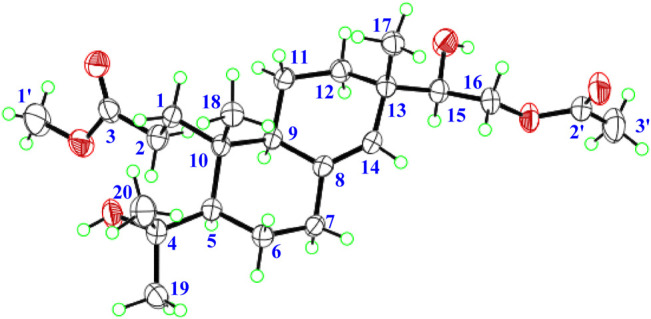
X-ray crystallographic of 1.

Biscognisecoisopimarate A (1) was obtained as colorless needle crystals. The cationic molecule peak at *m/z* 433.2563 [M + Na]^+^ (calcd. for C_23_H_38_O_6_Na, 433.2566) by HRESIMS indicated the molecular formula of 1 was C_23_H_38_O_6_ (5 degrees of unsaturation). The ^13^C NMR spectrum showed 23 carbon signals (Table 1). Combined with the DEPT-135 and HSQC experiment, these carbons can be categorized into four sp^2^ carbons, three sp^3^ quaternary carbons, three sp^3^ methine carbons, seven sp^3^ methylene carbons, and six methyl carbons. Four spin-coupling systems (H_2_-1–H_2_-2, H-5–H_2_-6–H_2_-7, H-9–H_2_-11–H_2_-12, and H-15–H_2_-16) were revealed by the analysis of ^1^H-^1^H COSY data of 1 (Figure 2). Combined with the ^1^H-^1^H COSY data, the HMBC correlations (Figure 2) from H-1b to C-5/ C-10, from H-2a/H-2b to C-3, from H-5 to C-10/C-18, from H-6a to C-4/C-10, from H-9 to C-7/C-8/C-10, from H-11b to C-8, from H-12a to C-17, from H-14 to C-9/C-12/C-13, from H-15 to C-13/C-14/C-17, from H-16a/H-16b to C-2', from H-17 to C-12/C-13/C-14, from H_3_-18 to C-4/C-5/C-19, from H_3_-19 to C-4/C-5/C-18, from H_3_-20 to C-1/C-5/C-9/C-10, from H-1' to C-3, and from H-3' to C-2 revealed the partial structure of 1. On the basis of the above analyses, the degrees of unsaturation, the molecular formula, and the chemical shift characteristics, the whole planar structure of 1 was established as shown in Figure 1. The single-crystal X-ray crystallographic analysis of 1 (Figure 3) confirmed the above deduction, and the values of the Flack parameter [0.10 (16)] and the Hooft parameter [0.09 (7)] allowed the absolute configuration of 1 as 5*R*, 9*S*, 10*R*, 13*S*, 15*R*.

**TABLE 1:** NMR data of 1-2 in CDCl_3_ (400 MHz for ^1^H; 100 MHz for ^13^C).

No	1		2	
	*δ*_C_, mult.	*δ*_H_ (*J* in Hz)^a^	*δ*_C_, mult.	*δ*_H_ (*J* in Hz)^a^
1	32.8, CH_2_	2.41, br t (13.2), Ha	30.8, CH_2_	2.30, Ha
		1.70, Hb		2.25, Hb
2	28.8, CH_2_	2.73, br t (13.2), Ha	27.0, CH_2_	1.93, Ha
		2.22, Hb		1.86, Hb
3	175.4, C		73.4, CH	3.82, dd (11.3, 4.7)
4	75.5, C		41.8, C	
5	52.0, CH	1.64	84.4, C	
6	25.5, CH_2_	1.60, Ha	192.5, C	
		1.46, Hb		
7	17.9, CH_2_	1.60, Ha	185.6, C	
		1.53, Hb		
8	139.8, C		130.8, C	
9	44.0, CH	1.94	149.6, C	
10	41.6, C		46.0, C	
11	35.5, CH_2_	2.25, Ha	125.0, CH	7.31, d (8.2)
		2.03, Hb		
12	29.2, CH_2_	1.53, Ha	134.5, CH	7.52, dd (8.2, 1.5)
		1.36, Hb		
13	38.1, C		148.3, C	
14	126.8, CH	5.29, s	126.1, CH	7.94, d (1.4)
15	77.1, CH	3.55, br d (8.7)	33.5, CH	2.95, *hept* (6.9)
16	66.0, CH_2_	4.32, dd (11.5, 1.5), Ha	23.6, CH_3_	1.26, d (6.9)
		3.94, dd (11.3, 9.2), Hb		
17	22.7, CH_3_	1.02, s	23.6, CH_3_	1.26, d (6.9)
18	27.6, CH_3_	1.26, s	23.0, CH_3_	1.27, s
19	34.6, CH_3_	1.27, s	16.6, CH_3_	1.43, s
20	18.2, CH_3_	0.91, s	29.2, CH_3_	1.39, s
1′	51.6, CH_3_	3.66, s		
2′	171.4, C			
3′	21.0, CH_3_	2.10, s		

a Indiscernible signals from overlap or complex multiplicity are reported without designating multiplicity.

**FIGURE 4:**
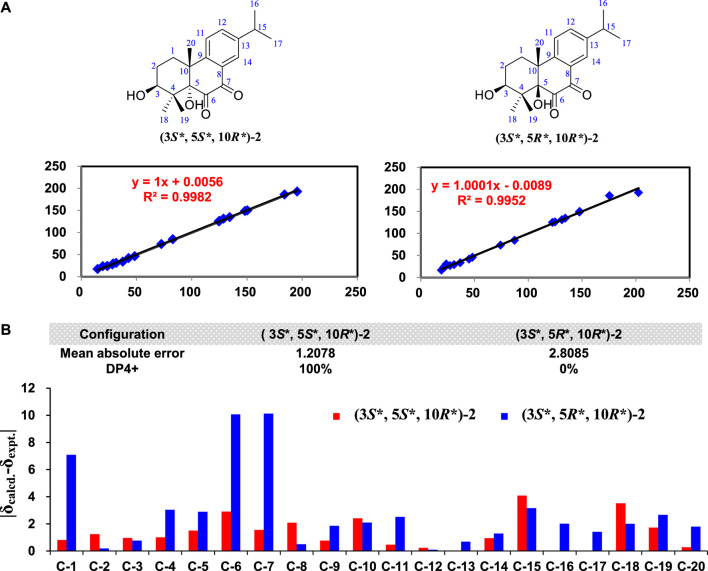
The ^13^C NMR calculation results of two plausible stereoisomers of 2 at B3LYP/6-31g+(d, p) level. **(A)** Linear coerrelation plots of calculated vs. experimental ^13^C NMR chemical shift values for two plausible stereoisomers. **(B)** Mean absolute errors between the calculated ^13^C NMR chemical shifts of of two plausible stereoisomers and the experimental ^13^C NMR data of 2 and DP4+ probability analysis.

**FIGURE 5:**
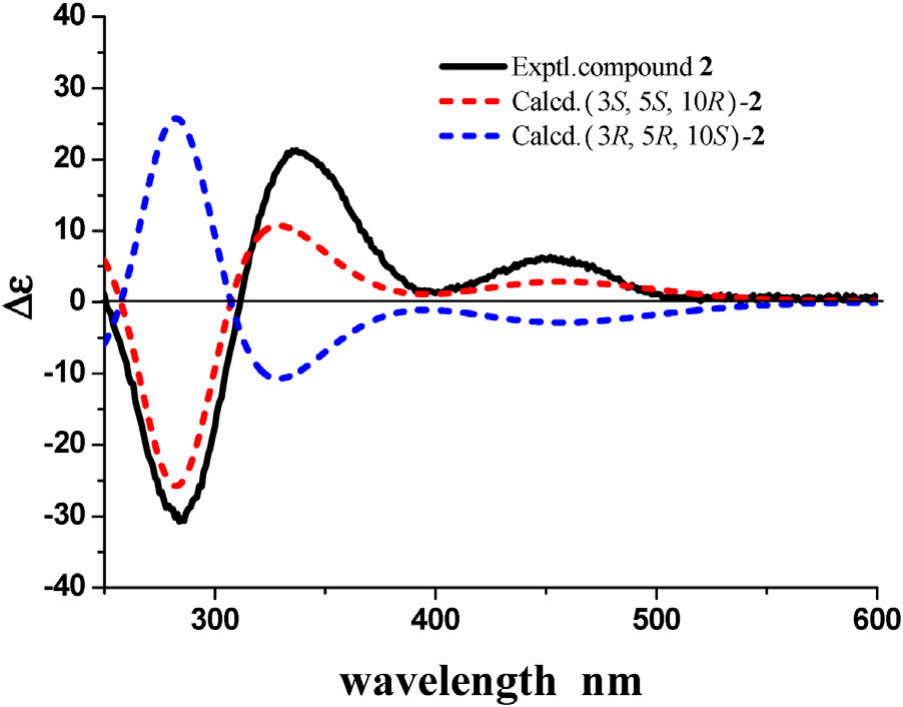
Experimental ECD spectra of 2 and calculated ECD spectra of (3S, 5S, 10R)-2 and (3R, 5R, 10S)-2(UV correction = −24 nm, band width *σ* = 0.3 eV.

3*β*-Hydroxyrickitin A (2) was obtained as a yellowish oil. In HR-ESI-MS, the protonated molecule peak at *m/z* 331.1915 [M + H]^+^ (calcd. for C_20_H_27_O_4_, 331.1909) indicated the molecular formula of C_20_H_26_O_4_ (8 degrees of unsaturation). Twenty carbon signals were observed in ^13^C NMR and DEPT 135 experiment, including eight sp^2^ carbons (192.5, 185.6, 149.6, 148.3, 134.5, 130.8, 126.1, 125.0), three sp^3^ quaternary carbons (84.4, 46.0, 41.8), two sp^3^ methine carbons (73.4, 33.5), two sp^3^ methylene carbons (30.8, 27.0), and five methyl carbons (29.2, 23.6, 23.6, 23.0, 16.6). All the proton resonances were associated to those of the directly attached carbon atoms through the HSQC experiment (Table 1). The analysis of the ^1^H-^1^H COSY experiment and the coupling values of protons revealed the presence of three isolated spin systems as shown in Figure 2. Combined with the ^1^H-^1^H COSY data, the HMBC correlations (Figure 2) from H-1a/H-1b to C-5/C-10/C-20, H-2a/H-2b to C-4/C-10, from H-11 to C-8/C-10/C-13, from H-12 to C-9/C-14, from H-14 to C-7/C-9/C-12, from H-15 to C-12/C-13/C-14, from H_3_-18 to C-3/C-4/C-19, from H_3_-19 to C-4/C-5/C-18, and from H_3_-20 to C-1/C-5/C-9/C-10, revealed the partial structure of 2. On the basis of the above analyses, the molecular formula, the degrees of unsaturation, and the chemical shifts of carbons, the planar structure of 2 was established as shown in Figure 1. The observed ROESY correlations between H_3_-20 and H-2b indicated that H_3_-20 and H-2b were on the same axial orientation in the cyclohexane ring (Supplementary Table S2 in Supporting Information). Detailed analysis of ^1^H-NMR signal of H-3 (3.82, dd, *J* = 11.3, 4.7 Hz) suggested that H-3 was on the axial orientation in the cyclohexane ring, adopting the opposite orientation as H-2b and H_3_-20. Meanwhile, the observed ROESY correlation between H-3 and H-1a in cyclohexane ring confirmed the above deduction (Supplementary Table S2 in Supporting Information). Therefore, the relative configurations of C-3 and C-10 were 3S*, 10R*. To determine the relative configurations of C-5 and C-10 in 2, the ^13^C chemical shifts calculations of (3S*, 5S*, 10R*)-2 and (3S*, 5R*, 10R*)-2 were calculated by a quantum chemical method at the B3LYP/6-311 + *g* (d,p) (Stanchev et al., 2012; Marell et al., 2014) level (see Supporting Information). Comparison between experimental and calculated data suggested that the calculated ^13^C chemical shifts of (3S*, 5S*, 10R*)-2 were similar to the experimental one (Figure 4; Supplementary Table S5) with a low mean absolute error value and a high DP4+ probability (100%) (Figure 4) (Grimblat et al., 2015). Therefore, the relative configuration of 2 was assigned as 3S*, 5S*, 10R*. The absolute configurations of C-3, C-5, and C-10 in 2 were determined by quantum chemical ECD calculation at B3LYP/6-311++*g* (2d,p) level. The predicted ECD curve of (3*S*, 5*S*, 10*R*)-**2** was similar to the experimental one (Figure 5; see Supporting Information). Therefore, the absolute configuration of 2 was established as 3*S*, 5*S*, and 10*R*.

**FIGURE 6:**
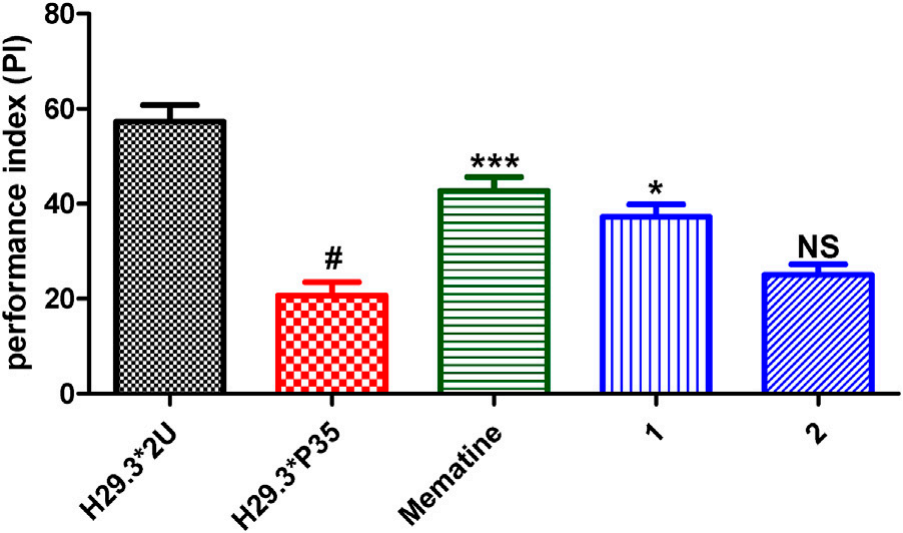
Performance index (PI) of AD flies fed with 1–2. The treated groups were AD flies treated with memantine or tested compounds (100 µM), and the control groups (H29.3*2U represents the normal flies, and H29.3*P35 represents the Ad flies were treated with corresponding volume of DMSO. Statistical analysis results of 1–2. Each value was expressed as means ± SEM, *n* = 8; #*p* < 0.001, significantly different from the AD group, *p < 0.005, significantly different from the AD group, and NS, no significantly different from the AD group; one way ANOVA.

In our work, a *seco*-isopimarane (1) and an abietane type (2) diterpenoids were obtained from *Biscogniauxia sp*. (No.71-10-1-1). This is the first report about diterpenoids from *Biscogniauxia sp*., which has enriched the structural diversity of this genus secondary metabolites. Furthermore, the bioassays (including short-term memory enhancement against AD, anti-inflammatory, and cytotoxic activities) of 1–2 have been carried out. The anti-Alzheimer’s disease (AD) activities of 1–2 were evaluated by AD fly model with memantine as the positive control. The result showed 1 exhibited short-term memory enhancement activity in AD flies (Figure 6). In addition, the anti-inflammatory assays of 1–2 were evaluated in LPS-stimulated RAW264.7 macrophages with hydrocortisone as the positive control. The results showed that compounds 1–2 showed cytotoxic effect on RAW264.7 cells. Therefore, their anti-inflammatory activities have not been tested. Furthermore, the cytotoxic activities of 1–2 against SW-480, MCF-7, HepG2, HeLa, and PANC-1 human cancer cell lines were evaluated by cell count kit 8 assay (CCK-8), using cisplatin as the positive control. However, none of them exhibited significant activity (IC_50_ > 40 μM).

## Materials and Methods

### General Experimental Procedures

The melting points were determined on an X-5 micro melting point apparatus (Beijing TECH Instrument Co. Ltd., Beijing, China) without corrected. UV data were recorded using a JASCO V-550 UV/vis spectrometer (Jasco International Co. Ltd., Tokyo, Japan). IR data were recorded on a JASCO FT/IR-480 plus spectrometer (Jasco International Co. Ltd., Tokyo, Japan). Optical rotations were measured on a JASCO P1020 digital polarimeter (Jasco International Co. Ltd., Tokyo, Japan). CD spectra were recorded in MeOH using a JASCO J-810 spectrophotometer (Jasco International Co. Ltd., Tokyo, Japan) at room temperature. ESI-MS spectra were performed on a Bruker amaZon SL mass spectrometer (Bruker Corporation, Boston, MA, United States). HRESIMS spectra were obtained on Waters Synapt G2 TOF mass spectrometer (Waters Corporation, Milford, MA, United States). 1D and 2D NMR spectra were acquired with Bruker AV 400 spectrometer (Bruker BioSpin Group, Faellanden, Switzerland) using the solvent signals (CDCl_3_: *δ*_H_ 7.26/ *δ*_C_ 77.0) as internal standards. Column chromatography (CC) was carried out on silica gel (200–300 mesh) (Qingdao Haiyang Chemical Group Corporation, Qingdao, China), and ODS (60–80 μm, YMC). TLC was performed on precoated silica gel plate (SGF254, 0.2 mm, Yantai Chemical Industry Research Institute, China). Analytical HPLC was performed on a Dionex HPLC system equipped with an Ultimate 3000 pump, an Ultimate 3000 diode array detector, an Ultimate 3000 column compartment, an Ultimate 3000 autosampler (Dionex, United States), and an Alltech (Grace) 2000ES evaporative light scattering detector (Alltech , KY, United States) using a Phenomenex Gemini C18 column (4.6 × 250 mm, 5 μm). Semi-preparative HPLC was carried out on Shimadzu LC-6AD system equipped with UV detectors, using a YMC-Pack ODS-A column (10.0 × 250 mm, 5 μm). Medium pressure liquid chromatography (MPLC) was equipped with a dual pump gradient system, a UV preparative detector, and a Dr Flash II fraction collector system (Shanghai Lisui E-Tech Co., Ltd.). Methanol (MeOH) was purchased from Yuwang Industrial Co. Ltd. (Yucheng, China). Acetonitrile (CH_3_CN) was obtained from Oceanpak Alexative Chemical Co. Ltd. (Gothenburg, Sweden). Cyclohexane, ethyl acetate (EtOAc), and chloroform (CHCl_3_) were analytical grade from Fine Chemical Co. Ltd. (Tianjin, China).

### Fungal Source, Fermentation, and Extraction

The strain numbered as 71-10-1-1 was isolated from the lichen *Usnea mutabilis* Stirt. collected from Zixi Mountain, Yunnan province, People’s Republic of China, in November, 2006. The strain was identified as *Biscogniauxia sp*. on the basis of the gene sequence analysis. The ribosomal internal transcribed spacer (ITS) and the 5.8S rRNA gene sequences (ITS1-5.8S-ITS2) of the strain have been deposited at GenBank as KX977400.

The fungal strain was cultured on slants of potato dextrose agar (PDA) at 25°C for 5 days. Agar plugs were used to inoculate nine Erlenmeyer flasks (250 ml), each containing 100 ml of potato dextrose broth (PDB). Fermentation was carried out in 60 Erlenmeyer flasks (500 ml), each containing 70 g of rice. Distilled H_2_O (105 ml) was added to each flask, and the rice was soaked overnight before autoclaving at 120°C for 30 min. After cooling to room temperature, each flask was inoculated with 5.0 ml of the spore inoculum and incubated at room temperature for 50 days.

### Isolation of Secondary Metabolites

The culture was extracted thrice with EtOAc, and the organic solvent was evaporated to dryness under vacuum to afford a crude extract (109.5 g). Then the crude extract was subjected to silica gel CC (4 × 15 cm) using cyclohexane-MeOH (100:0 and 0:100, v/v) to afford cyclohexane extract (C, 52.2 g) and MeOH extract (w, 49.8 g). The MeOH extract (w, 49.8 g) was separated by ODS CC (4 × 30 cm) eluting with MeOH–H_2_O elution (20:80, 50:50, 70:30 and 100:0, v/v) to afford 4 fractions (w1–w4). The fraction w3 (8.5 g) was further subjected to MPLC on ODS CC (4 × 45 cm) eluted with a gradient of MeOH-H_2_O (20:80 to 100:0, v/v ) with flow rate 20 ml/min for 470 min to afford 13 major fractions (Fr. w3-1–w3-13). Subfraction w3-8 (1457.6 mg) was subjected to silica gel CC using cyclohexane-ethyl acetate (100:0 and 0:100, v/v) to afford subfractions w3-8-1–w3-8-12. Subfraction w3-8-7 (78.1 mg) was subjected to semi-preparative HPLC, using MeCN–H_2_O (35:65, v/v) at a flow rate of 3 ml/min to yield 1 (*t*_R_: 60.3 min, 6.6 mg). Subfraction w3-8-6 (103.5 mg) was subjected to semi-preparative HPLC, using MeCN–H_2_O (35: 65, v/v) at a flow rate of 3 ml/min to yield 2 (*t*_R_: 54.3 min, 5.5 mg).

### Biscognisecoisopimarate A (1)

Colorless needle crystal; mp 164–166°C; [α]^27.5^ D 52.2 (*c* 0.5, MeOH); UV (MeOH) λmax (logε) 207 (3.78), 252 (1.87) nm; IR (KBr) νmax 3448, 2872, 1730, 1708, 1651, 1457, 1264, 1034 cm^−1^; ESI-MS (positive) *m/z* 433 [M + Na]^+^; HRESIMS (positive) *m/z* 433.2563 [M + Na]^+^ (calcd. for C_23_H_38_O_6_Na, 433.2566); ^1^H and ^13^C NMR see Table 1.

### 3*β*-Hydroxyrickitin A (2)

Yellowish oil; [α]^27.5^ D 130.1 (*c* 0.5, MeOH); UV (MeOH) *λ*_max_ (log *ε*) 214 (4.05), 284 (3.71) nm; CD *λ*_nm_ (∆*ε*) (*c* 1.6 × 10^–4^ mol/L, CH_3_Cl) 284 (–30.70), 336 (+21.13), 453 (+6.03) nm; IR (KBr) *ν*_max_ 3449, 2955, 2929, 1734, 1691, 1612, 1461, 1382, 1307, 1065 cm^−1^; ESI-MS (positive) *m/z* 331 [M + H]^+^, *m/z* 353 [M + Na]^+^; HRESIMS (positive) *m/z* 331.1915 [M + H]^+^ (calcd. for C_20_H_27_O_4_, 331.1909); ^1^H and ^13^C NMR data see Table 1.

### X-Ray Crystallographic Analysis

Crystallographic data of 1: Upon crystallization from MeOH using the vapor diffusion method, colorless needle-like crystals of 1 were obtained. Data were collected using a Sapphire CCD with graphite monochromated Cu Kα radiation, *λ* = 1.54184 Åat 173.00 (10) K. Crystal data: C_23_H_38_O_6_, *M* = 410.53, space group monoclinic, *P* 2_1_; unit cell dimensions were determined to be *a* = 12.2840 (2) Å, *b* = 6.04757 (14) Å, *c* = 15.0642 (3) Å, *α* = 90.00 °, *β* = 95.7465 (18) °, *γ* = 90.00 °, *V* = 1113.47 (4) Å^3^, *Z* = 2, *Dx* = 1.224 g/cm^3^, *F* (000) = 448.0, *μ* (Cu Kα) = 0.703 mm^−1^. 17244 reflections were collected to *θ*_max_ = 62.84°, in which 3365 independent unique reflections (*R*_int_ = 0.0299, *R*_sigma_ = 0.0210) were used in all calculations. The structure was solved by direct methods using the SHELXS program, and refined by the SHELXL program and full-matrix least-squares calculations.^5^ In the structure refinements, hydrogen atoms were placed on the geometrically ideal positions by the “ride on” method. The final refinement gave *R*_1_ = 0.0300 (I > 2σ(I)), _W_ *R*_2_ = 0.0768 (all data), *S* = 1.058, Flack = 0.10 (16) and Hooft = 0.09 (7). Crystallographic data for 1 have been deposited in the Cambridge Crystallographic Data Center as supplementary publication no. CCDC 2099938. Copies of the data can be obtained, free of charge, on application to the Director, CCDC, 12 Union Road, Cambridge CB2 1EZ, United Kingdom (fax: +44-(0)1223-336033, or e-mail: deposit@ccdc.cam.ac.uk).

### Short-Term Memory Assays of 1–2 on the AD Fly Model

Fly Stock. w1118 (isoCJ1) is an isogenic line used as a control in all of the experiments. In the experiment, we named this stock “2U” for convenience. Expression of A*β*_42_ (UAS-A*β*_42_; referred to as H29.3) was driven by a widespread neuronal-expressing Gal4 line, elav-GAL4c155 (P35). A behavioral assay was made from the first generation of the cross between H29.3*P35 (AD flies).

Fly Culture. All flies were fed at 24°C, 40–60% RH (relative humidity). On day 2, newly hatched H29.3*2U male flies and AD male flies were selected and were put into vials (each vial contains about 100 flies). These flies were placed at 29°C, 40 ± 15% RH during the drug feeding process. The flies were transferred to new vials after 4 h of drug feeding from day 2 to day 8. All flies were placed at 29°C, 40 ± 15% RH.

Drug Feeding Schedule. Drugs (compounds 1–2 and memantine) were prepared on the first day of eclosion, and the drug feeding was implemented on day 2. The original concentration of compounds was 10 mM, and the final concentration of compounds was 100 μM. These compounds were dissolved in DMSO. For each performance index, 2 vials of flies were fed with 50 μL of the resulting solution for 7 days (e.g., from day 2 to day 8).

Pavlovian Olfactory Learning. Briefly, in the training period, a group of 100 flies was put into a training tube decorated with copper grids. These flies would be orderly exposed to two odors (octanol or methyl cyclohexanol) for 1 min, who were subsequently surrounded by fresh air for 45 s. These flies would be subjected to electric shock for 1 min when they smell the first odor. In the test period, these trained flies would be immediately transported to the choice point, where the T-maze diverged into two containers containing two odors. The flies were allowed to choose one odor between the two for 2 min. Then the number of flies in two containers would be counted. The parameter of PI (performance index) was used for evaluating flies’ memory behavior. PI = 0 represents these flies cannot memorize the link between electric shock and odors. On the contrary, PI = 100% represents all the flies can avoid electric shock according to odors. Note: The experiment was carried out in a room with 25°C, 70% RH.

Statistical Analysis. Data were analyzed and graphs were also plotted (Figure 6) with the program Graph Pad 5.03.

### Anti-Inflammatory Assays of 1–2

Cell culture and treatment. RAW264.7 murine macrophage cell line was obtained from American Type Culture Collection (ATCC, Manassas, VA, United States). Cells were cultured in DMEM medium (Gibco, Life technologies, CA, United States) supplemented with 10% heat-inactivated FBS (Excell biotech Co. Ltd., Shanghai, China), 2 mM L-glutamine (Gibco), 100 U/ml penicillin and 100 μg/ml streptomycin (Gibco) at 37°C with 5% CO_2_. The cells were cultured for 2–3 days to reach the logarithmic phase and then used for experiments. The cells were treated with tested compounds and positive control (hydrocortisone (Sigma, United States)) at different concentrations (5, 10, 20, 40, 80 µM) and then stimulated with LPS (Sigma, United States) for the incubated time. The stock solutions of tested compounds were prepared in DMSO, and the final concentration of DMSO was less than 0.1%.

Cell viability assay. The cytotoxic effect of compounds 1–2 on RAW264.7 cells was evaluated by MTT assay. RAW264.7 cells were dispensed in 96-well plate at a density of 1 × 10^5^ cell per well incubated at 37°C for 24 h incubation, cells were treated with the tested agents for the indicated periods of time. Then, 20 µl aliquot of 0.5% MTT (Sigma, MA, United States) solution was added to each well followed by 4 h incubation. The culture medium was removed, and the formazan precipitates were dissolved with 150 µl of DMSO. The optical densities (OD) at 570 nm were measured with a ELISA reader (Thermo Fisher Scientific, Franklin, MA, United States). Compounds 1–2 showed no cytotoxic effect on RAW264.7 cells at different concentrations (5, 10, 20, 40, 80 µM).

### Cytotoxicity Assays of 1-2

Five human cancer cell lines (SW-480, MCF-7, HepG2, HeLa, and PANC-1) were used in the cytotoxicity assays. All the cells were cultured in DMEM medium (Gibco, Life technologies, United States), supplemented with 10% fetal bovine serum (Gibco, Life technologies, United States), in 5% CO_2_ at 37 °C. The cytotoxicity assay was performed using the CCK-8 (Dojindo Laboratories, Kumamoto, Japan) assay according to the manufacturer’s protocol. Briefly, 1 × 10^4^ cells were seeded into each well of the 96 well plate and treated with different concentrations (2.5–40 μM) of the compounds with cisplatin (Sigma, MA, United States) as the positive control for 48 h. Then, CCK-8 solution (10 μl) was added to each well and incubated for 2 h at 37°C. The absorbance of the samples at 450 nm was measured using a microtiter plate reader (TriStar LB941, Berthold technologies, Germany). The 50% inhibitory concentration (IC_50_) of each compound was calculated using a weighted regression of the plot.

## Data Availability

The crystallographic dataset generated for this study can be found in the Cambridge Crystallographic Data Centre under the CCDC number 2099938. All other datasets generated for this study are included in the article/Supplementary Material.
