# Supplementary material for: Two New Diterpenoids from Biscogniauxia sp. and Their Activities
Source: Front Chem. 2021 Sep 1;9:749272. doi: 10.3389/fchem.2021.749272 (PMC8440928; doi:10.3389/fchem.2021.749272)
Supplement: Supplementary file 1 [file DataSheet1.doc]

Supplementary Material

List of Supporting Information

[**1. NMR data assignments of 1‒2 2**](#__RefHeading___Toc79937024)

[**2. ^13^C chemical shifts calculation of 2 4**](#__RefHeading___Toc79937025)

[**3. Quantum chemical ECD calculation of 2 8**](#__RefHeading___Toc79937026)

[**4. The 1D and 2D NMR spectra of 1−2 11**](#__RefHeading___Toc79937027)

[**5. The screenshots of the checkCIF file of 1 17**](#__RefHeading___Toc79937028)

# NMR data assignments of 1‒2

**Table S1** NMR data of **1** in CDCl_3_ (400 MHz for ^1^H; 100 MHz for ^13^C)

| No. | *δ*_C_, mult | *δ*_H_ (*J* in Hz) ^a^ | ^1^H-^1^H COSY | HMBC |
| --- | --- | --- | --- | --- |
| 1 | 32.8, CH_2_ | 2.41, br t (13.2), Ha | 1b, 2a, 2b | 2 |
|  |  | 1.70, Hb | 1a, 2a, 2b | 2, 5, 10 |
| 2 | 28.8, CH_2_ | 2.73, br t (13.2), Ha | 1a, 1b, 2b | 1, 3 |
|  |  | 2.22, Hb | 1a, 1b, 2a | 1, 3 |
| 3 | 175.4, C |  |  |  |
| 4 | 75.5, C |  |  |  |
| 5 | 52.0, CH | 1.64 | 6a, 6b | 4, 7, 10, 18 |
| 6 | 25.5, CH_2_ | 1.60, Ha | 5, 6b, 7a, 7b | 4, 7, 10 |
|  |  | 1.46, Hb | 5, 6a, 7a, 7b |  |
| 7 | 17.9, CH_2_ | 1.60, Ha | 6a, 6b, 7b |  |
|  |  | 1.53, Hb | 6a, 6b, 7a |  |
| 8 | 139.8, C |  |  |  |
| 9 | 44.0, CH | 1.94 | 11a, 11b | 7, 8, 10, 14, 20 |
| 10 | 41.6, C |  |  |  |
| 11 | 35.5, CH_2_ | 2.25, Ha | 9, 11b, 12a, 12b |  |
|  |  | 2.03, Hb | 9, 11a, 12a, 12b | 8 |
| 12 | 29.2, CH_2_ | 1.53, Ha | 11a, 11b, 12b | 17 |
|  |  | 1.36, Hb | 11a, 11b, 12a |  |
| 13 | 38.1, C |  |  |  |
| 14 | 126.8, CH | 5.29, s |  | 9, 11, 12, 13, 15 |
| 15 | 77.1, CH | 3.55, br d (8.7) | 16a, 16b | 12, 13, 14, 16, 17 |
| 16 | 66.0, CH_2_ | 4.32, dd (11.5, 1.5), Ha | 15, 16b | 2′ |
|  |  | 3.94, dd (11.3, 9.2) , Hb | 15, 16a | 15, 2′ |
| 17 | 22.7, CH_3_ | 1.02, s |  | 12, 13, 14, 15 |
| 18 | 27.6, CH_3_ | 1.26, s |  | 4, 5, 19 |
| 19 | 34.6, CH_3_ | 1.27, s |  | 4, 5, 18 |
| 20 | 18.2, CH_3_ | 0.91, s |  | 1, 5, 9, 10 |
| 1′ | 51.6, CH_3_ | 3.66, s |  | 3 |
| 2′ | 171.4, C |  |  |  |
| 3′ | 21.0, CH_3_ | 2.10, s |  | 2′ |

*^a^* Indiscernible signals owing to overlapping or having complex multiplicity are reported without designating multiplicity.

**Table S2** NMR data of **2** in CDCl_3_ (400 MHz for ^1^H; 100 MHz for ^13^C)

| No. | *δ*_C_, mult | *δ*_H_ (*J* in Hz) ^a^ | ^1^H-^1^H COSY | HMBC | ROESY |
| --- | --- | --- | --- | --- | --- |
| 1 | 30.8, CH_2_ | 2.30, Ha | 1b, 2a, 2b | 2, 3, 5, 10, 20 | 3 |
|  |  | 2.25, Hb | 1a, 2a, 2b | 2, 3, 5, 10, 20 | 11, 12, 20 |
| 2 | 27.0, CH_2_ | 1.93, Ha | 1a, 1b, 2b, 3 | 1, 3, 4, 10 | 3 |
|  |  | 1.86, Hb | 1a, 1b, 2a, 3 | 1, 3, 4, 10 | 19, 20 |
| 3 | 73.4, CH | 3.82, dd (11.3, 4.7) | 2a, 2b | 18, 19 | 1a, 2a, 18 |
| 4 | 41.8, C |  |  |  |  |
| 5 | 84.4, C |  |  |  |  |
| 6 | 192.5, C |  |  |  |  |
| 7 | 185.6, C |  |  |  |  |
| 8 | 130.8, C |  |  |  |  |
| 9 | 149.6, C |  |  |  |  |
| 10 | 46.0, C |  |  |  |  |
| 11 | 125.0, CH | 7.31, d (8.2) | 12 | 7, 8, 10, 12, 13 | 1b |
| 12 | 134.5, CH | 7.52, dd (8.2, 1.5) | 11,14 | 9, 11, 14, 15 | 1b, 15 |
| 13 | 148.3, C |  |  |  |  |
| 14 | 126.1, CH | 7.94, d (1.4) | 12 | 7, 9, 12, 15 |  |
| 15 | 33.5, CH | 2.95, *hept* (6.9) | 16, 17 | 12, 13, 14, 16, 17 | 12 |
| 16 | 23.6, CH_3_ | 1.26, d (6.9) | 15 | 13, 15, 17 |  |
| 17 | 23.6, CH_3_ | 1.26, d (6.9) | 15 | 13, 15, 16 |  |
| 18 | 23.0, CH_3_ | 1.27, s |  | 3, 4, 5, 19 | 3 |
| 19 | 16.6, CH_3_ | 1.43, s |  | 3, 4, 5, 18 | 2b |
| 20 | 29.2, CH_3_ | 1.39, s |  | 1, 5, 9, 10 | 1b, 2b |

*^a^* Indiscernible signals owing to overlapping or having complex multiplicity are reported without designating multiplicity.

# ^13^C chemical shifts calculation of 2

The molecules of (3*S**, 5*S**, 10*R**)-**2** and (3*S**, 5*R**, 10*R**)-**2** were converted into SMILES codes before their initial 3D structures were generated with CORINA version 3.4. Conformer databases were generated in CONFLEX version 7.0 using the MMFF94s force-field, with an energy window for acceptable conformers (ewindow) of 5 kcal/mol^-1^ above the ground state, a maximum number of conformations per molecule (maxconfs) of 100, and an RMSD cutoff (rmsd) of 0.5Å. Then each conformer of the acceptable conformers was optimized with HF/6-31G(d) method in Gaussian09. ^1^ Further optimization at the APFD/6-31G(d) level determined the dihedral angles. From this, 12 lowest energy conformers of (3*S**, 5*S**, 10*R**)-**2** and 2 lowest energy conformers of (3*S**, 5*R**, 10*R**)-**2** were obtained. The optimized conformers were used for the NMR calculations, which were performed in B3LYP/6-311+g(d,p) level. The solvent effects were taken into account by the polarizable-conductor calculation model (PCM, chloroform as the solvent). The calculated ^13^C chemical shifts were summed by the boltzmann weighting. The calculated results of three methods [B3LYP/6-311+g (d, p) was shown in **Table S5**. The comparison was judged by mean absolute error and DP4+ probability. ^2^

**Table S3** Conformers distribution of (3*S*, 5*S*, 10*R*)-**2** in solvated models calculations at the APFD/6-31G (d)

| Conformers | Contribution % | Conformers | Contribution % |
| --- | --- | --- | --- |
| 1 | 18.02 | 7 | 5.87 |
| 2 | 17.80 | 8 | 5.69 |
| 3 | 11.81 | 9 | 5.18 |
| 4 | 11.50 | 10 | 4.89 |
| 5 | 6.71 | 11 | 2.97 |
| 6 | 6.64 | 12 | 2.90 |

**Figure S1.** Most stable conformers of (3*S*, 5*S*, 10*R*)-**2** (the relative populations are in parentheses)

**Table S4** Conformers distribution of (3*S*, 5*R*, 10*R*)-**2** in solvated models calculations at the APFD/6-31G (d)

| Conformers | Contribution % |
| --- | --- |
| 1 | 50.83 |
| 2 | 49.17 |

**Figure S2.** Most stable conformers of (3*S*, 5*R*, 10*R*)-**2** (the relative populations are in parentheses)

**Table S5 The ^13^C chemical shifts of two isomers of 2 calculated in the level of B3LYP/6-311+g (d, p) and the experimental data of 2**

|  | Expt. | (3*S**, 5*S**, 10*R**)-**2** |  | (3*S**, 5*R**, 10*R**)-**2** |  |
| --- | --- | --- | --- | --- | --- |
| 1 | 30.8 | 31.6136 | 0.8136 | 23.7159 | 7.0841 |
| 2 | 27.0 | 28.2405 | 1.2405 | 27.1810 | 0.1810 |
| 3 | 73.4 | 72.4462 | 0.9538 | 74.1662 | 0.7662 |
| 4 | 41.8 | 42.8021 | 1.0021 | 44.8397 | 3.0397 |
| 5 | 84.4 | 82.8932 | 1.5068 | 87.2832 | 2.8832 |
| 6 | 192.5 | 195.4021 | 2.9021 | 202.5671 | 10.0671 |
| 7 | 185.6 | 184.0500 | 1.5500 | 175.4722 | 10.1278 |
| 8 | 130.8 | 128.7264 | 2.0736 | 131.2974 | 0.4974 |
| 9 | 149.6 | 150.3561 | 0.7561 | 147.7518 | 1.8482 |
| 10 | 46.0 | 48.4014 | 2.4014 | 48.0957 | 2.0957 |
| 11 | 125.0 | 124.5429 | 0.4571 | 122.4931 | 2.5069 |
| 12 | 134.5 | 134.2673 | 0.2327 | 134.4095 | 0.0905 |
| 13 | 148.3 | 148.2850 | 0.0150 | 147.6185 | 0.6815 |
| 14 | 126.1 | 125.1583 | 0.9417 | 124.8144 | 1.2856 |
| 15 | 33.5 | 37.5778 | 4.0778 | 36.6524 | 3.1524 |
| 16 | 23.6 | 23.5709 | 0.0291 | 21.5946 | 2.0054 |
| 17 | 23.6 | 23.6020 | 0.0020 | 22.1899 | 1.4101 |
| 18 | 23.0 | 19.4827 | 3.5173 | 25.0040 | 2.0040 |
| 19 | 16.6 | 14.8835 | 1.7165 | 19.2547 | 2.6547 |
| 20 | 29.2 | 28.9239 | 0.2761 | 30.9892 | 1.7892 |
| Mean absolute error |  |  | 1.2078 |  | 2.8085 |


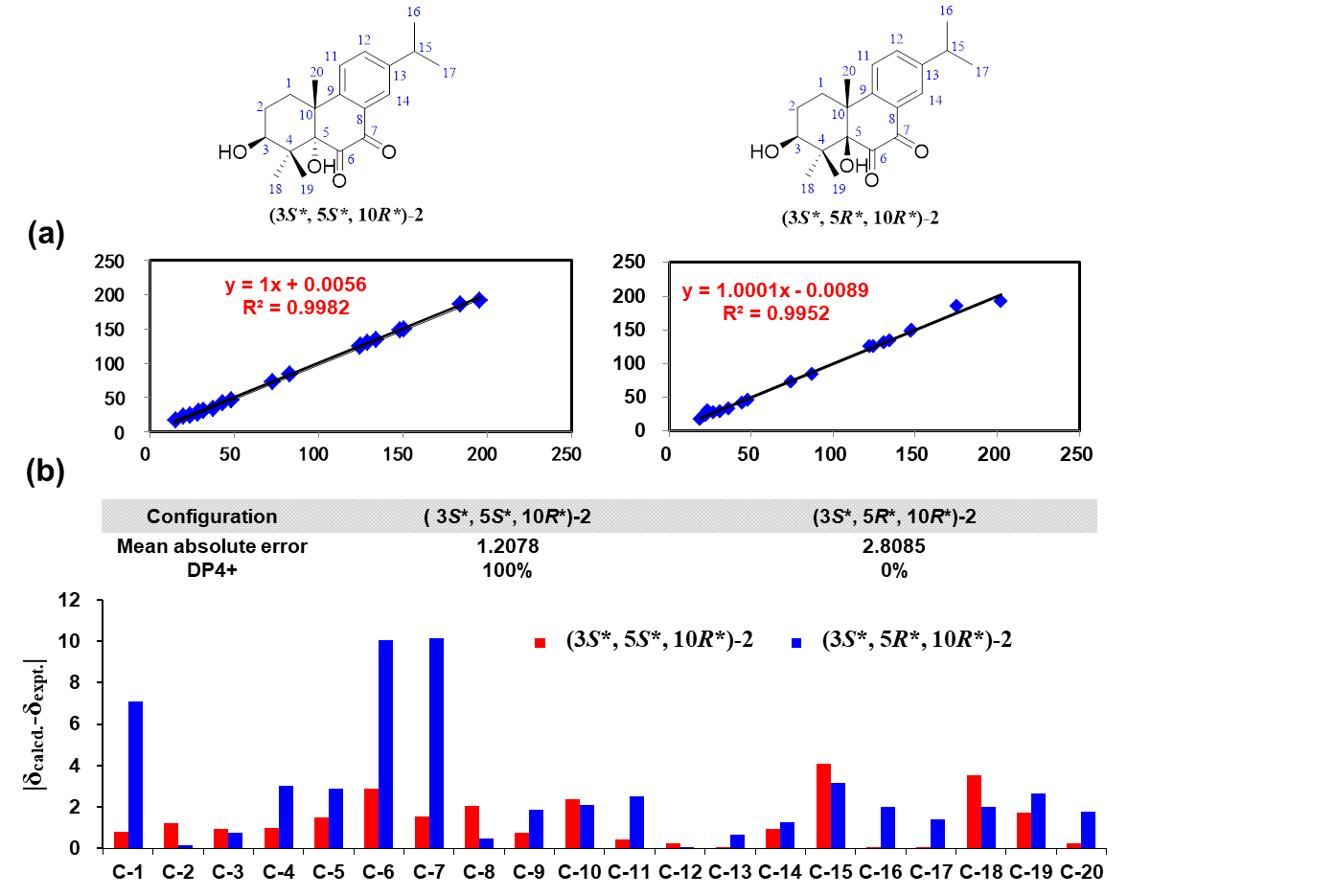


**Figure S3**. The ^13^C NMR calculation results of two plausible stereoisomers of **2** at B3LYP/6-31g+(d,p) level. (a) Linear correlation plots of calculated vs. experimental 1^3^C NMR chemical shift values for two plausible stereoisomers. (b) Mean absolute errors between the calculated ^13^C NMR chemical shifts of two plausible stereoisomers and the experimental ^13^C NMR data of **2**, and DP4+ probability analysis.

# Quantum chemical ECD calculation of 2

The molecules of (3*S**, 5*S**, 10*R**)-**2** and (3*R**, 5*R**, 10*S**)-**2** were converted into SMILES codes before their initial 3D structures were generated with CORINA version 3.4. Conformer databases were generated in CONFLEX version 7.0 using the MMFF94s force-field, with an energy window for acceptable conformers (ewindow) of 5 kcal mol^-1^ above the ground state, a maximum number of conformations per molecule (maxconfs) of 100, and an RMSD cutoff (rmsd) of 0.5Å. Then each conformer of the acceptable conformers was optimized with HF/6-31G (d) method in Gaussian09. ^1^ Further optimization at the B3LYP/6-31G (d) level determined the dihedral angles. After that, 12 lowest energy conformers were obtained. The optimized conformers were taken for the ECD calculations, which were performed with Gaussian09 (B3LYP/6-311++G (2d, p)). The solvent effects were taken into account by the polarizable-conductor calculation model (PCM, chloroform as the solvent). Comparisons of the experimental and calculated spectra were done with the software SpecDis.^3, 4^ It was also used to apply a UV shift to the ECD spectra, Gaussian broadening of the excitations, and Boltzmann weighting of the spectra.

**Table S6** Conformers distribution of (3*S*, 5*S*, 10*R*)-**2** in solvated models calculations at the B3LYP/6-31G (d)

| Conformers | Contribution % | Conformers | Contribution % |
| --- | --- | --- | --- |
| 1 | 16.91 | 7 | 5.52 |
| 2 | 15.49 | 8 | 5.18 |
| 3 | 11.40 | 9 | 4.99 |
| 4 | 10.69 | 10 | 4.53 |
| 5 | 10.01 | 11 | 3.15 |
| 6 | 9.31 | 12 | 2.81 |

**Figure S4.** Most stable conformers of (3*S*, 5*S*, 10*R*)-**2** (the relative populations are in parentheses)

**Figure S5.** Experimental ECD spectra of **2** and calculated ECD spectra of (3*S*, 5*S*, 10*R*)-**2** and (3*R*, 5*R*, 10*S*)-**2** (UV correction = -24 nm, band width σ = 0.3 eV)

**References**

1. Frisch, M. J.; Trucks, G. W.; Schlegel, H. B.; Scuseria, G. E.; Robb, M. A.; Cheeseman, J. R.; Scalmani, G.; Barone, V.; Mennucci, B.; Petersson, G. A.; Nakatsuji, H.; Caricato, M.; Li, X.; Hratchian, H. P.; Izmaylov, A. F.; Bloino, J.; Zheng, G.; Sonnenberg, J. L.; Hada, M.; Ehara, M.; Toyota, K.; Fukuda, R.; Hasegawa, J.; Ishida, M.; Nakajima, T.; Honda, Y.; Kitao, O.; Nakai, H.; Vreven, T.; Montgomery, J. A., Jr.; Peralta, J. E.; Ogliaro, F.; Bearpark, M.; Heyd, J. J.; Brothers, E.; Kudin, K. N.; Staroverov, V. N.; Kobayashi, R.; Normand, J.; Raghavachari, K.; Rendell, A.; Burant, J. C.; Iyengar, S. S.; Tomasi, J.; Cossi, M.; Rega, N.; Millam, J. M.; Klene, M.; Knox, J. E.; Cross, J. B.; Bakken, V.; Adamo, C.; Jaramillo, J.; Gomperts, R.; Stratmann, R. E.; Yazyev, O.; Austin, A. J.; Cammi, R.; Pomelli, C.; Ochterski, J. W.; Martin, R. L.; Morokuma, K.; Zakrzewski, V. G.; Voth, G. A.; Salvador, P.; Dannenberg, J. J.; Dapprich, S.; Daniels, A. D.; Farkas, Ö.; Foresman, J. B.; Ortiz, J. V.; Cioslowski, J.; Fox, D. J. Gaussian 09, Revision C1; Gaussian, Inc., Wallingford CT, 2010.
2. Grimblat, N.; Zanardi, M. M.; Sarotti, A.M. J. Org. Chem. 2015, 80, 12526–12534.
3. Bruhn, T.; Schaumlöffel, A.; Hemberger, Y; Bringmann, G; Version 1.61 ed.; University of Würzburg: Würzburg, Germany, 2013.

4. Bruhn, T.; Schaumlöffel, A.; Hemberger, Y; Bringmann, G. Chirality 2013, 25, 243–249.

# 4. The 1D and 2D NMR spectra of 1−2


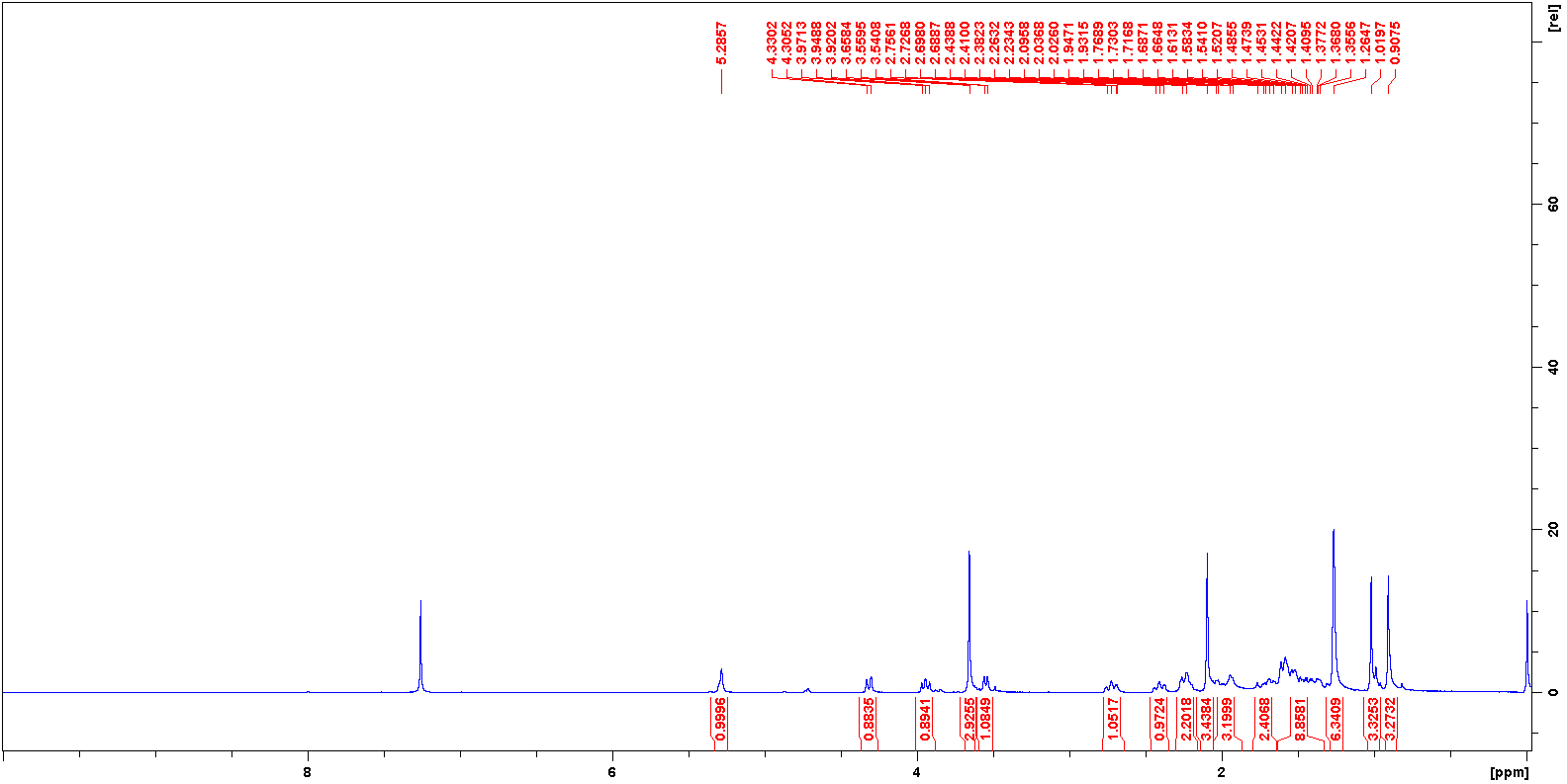


**Figure S6.** ^1^H NMR (400 MHz, CDCl_3_) spectrum for **1**


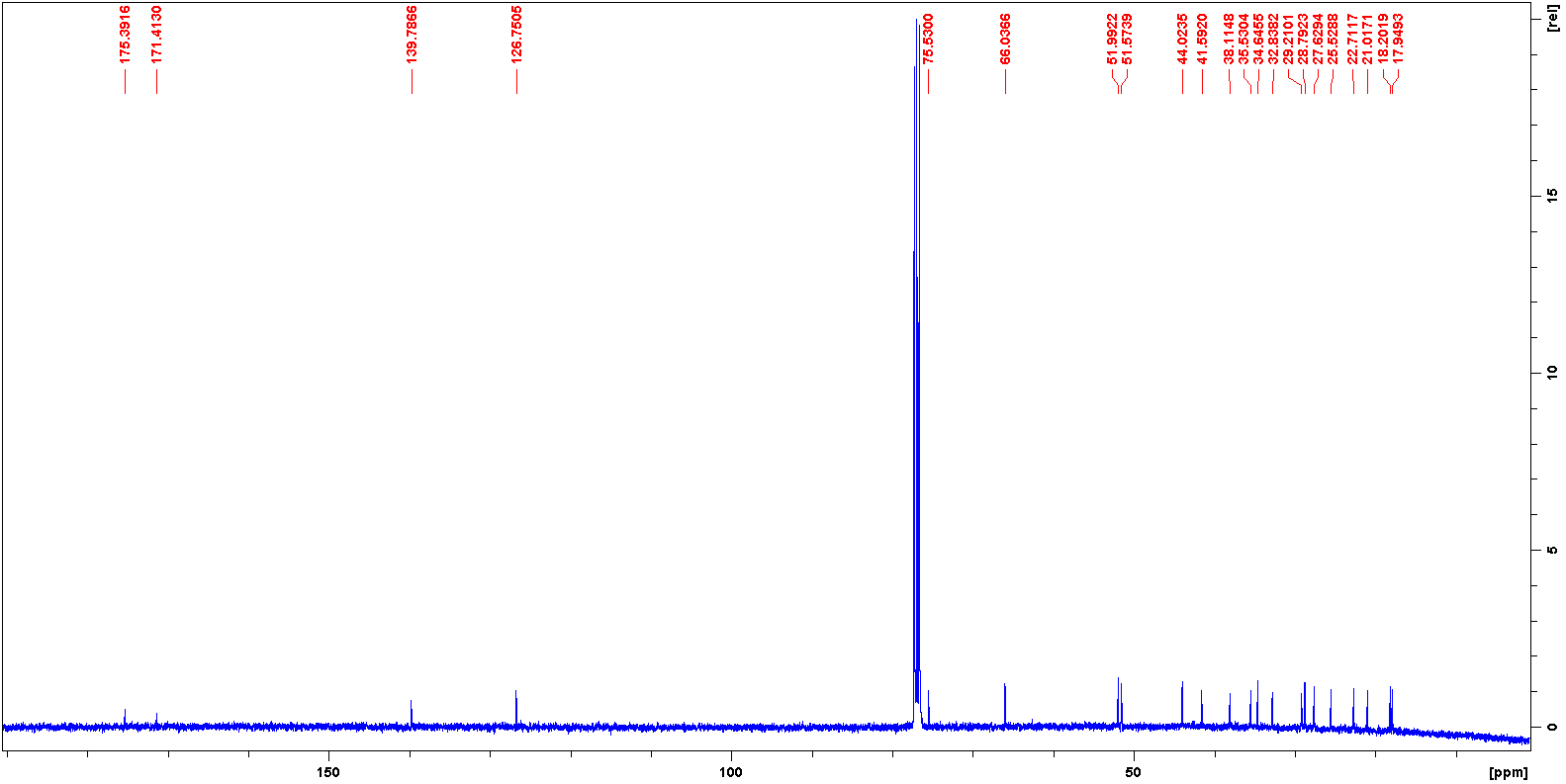


**Figure S7.** ^13^C NMR (100 MHz, CDCl_3_) spectrum for **1**


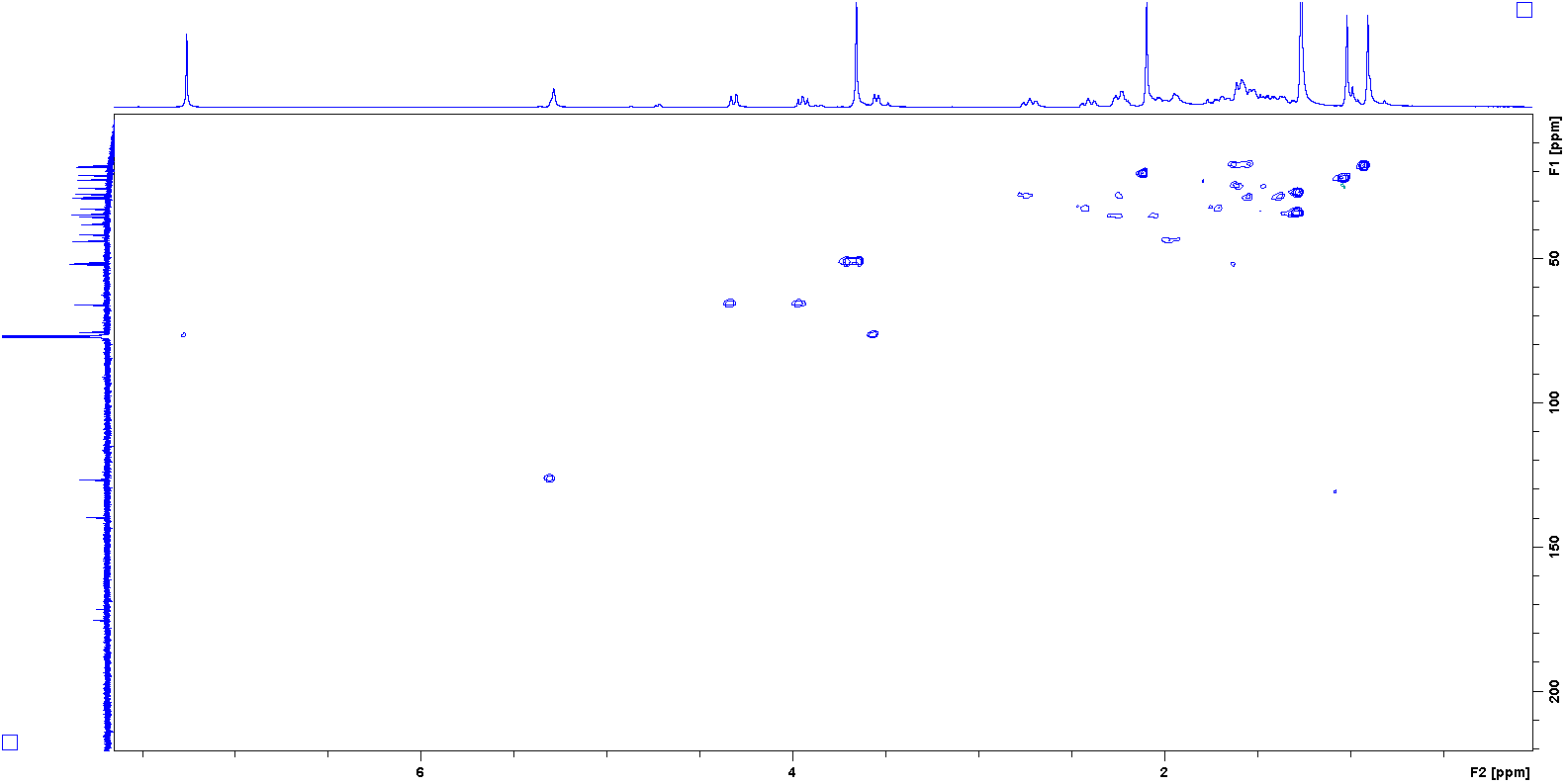


**Figure S8.** HSQC (400 MHz, CDCl_3_) spectrum for **1**


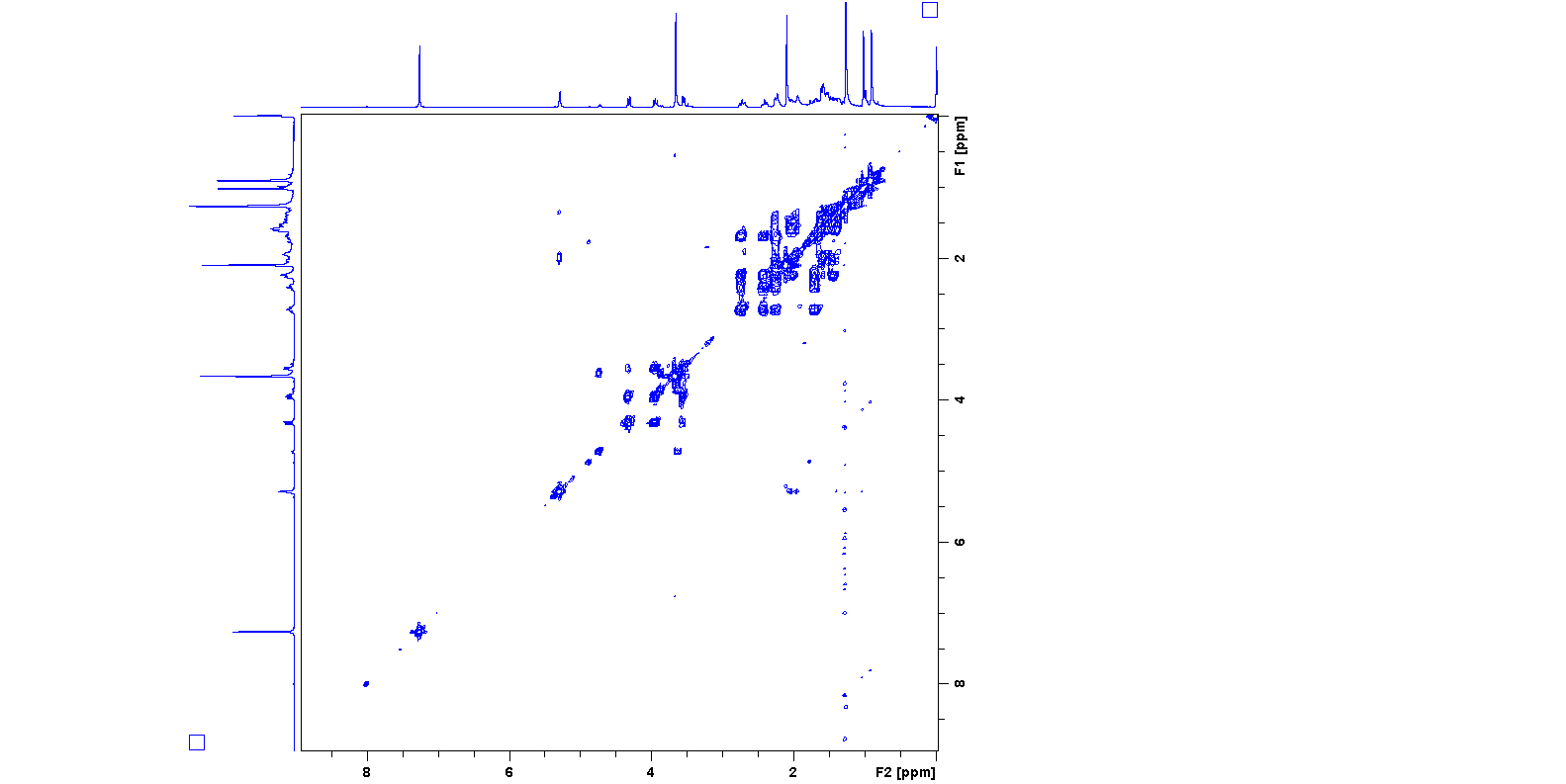


**Figure S9.** ^1^H-^1^H COSY (400 MHz, CDCl_3_) spectrum for **1**


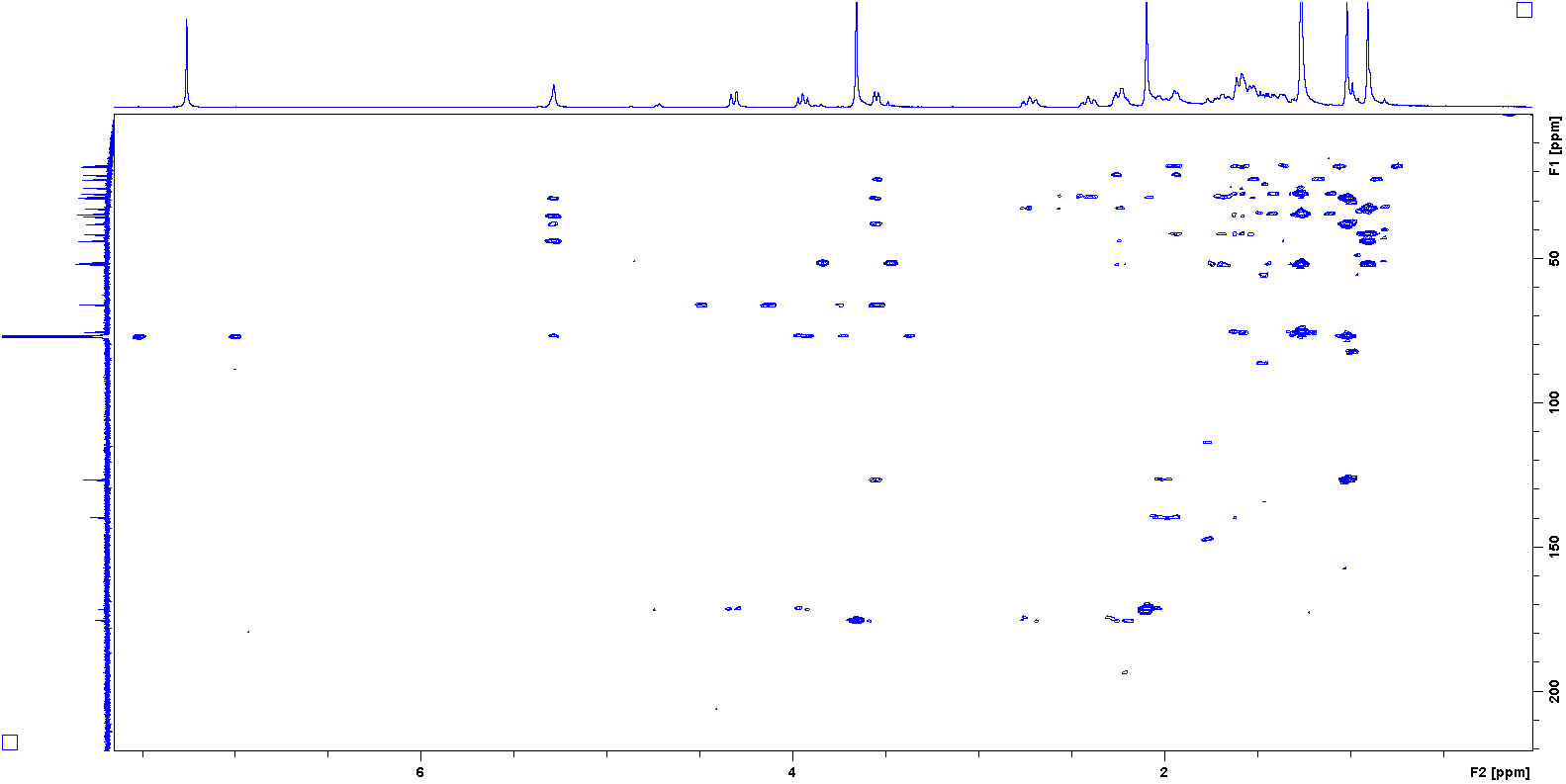


**Figure S10.** HMBC (400 MHz, CDCl_3_) spectrum for **1**


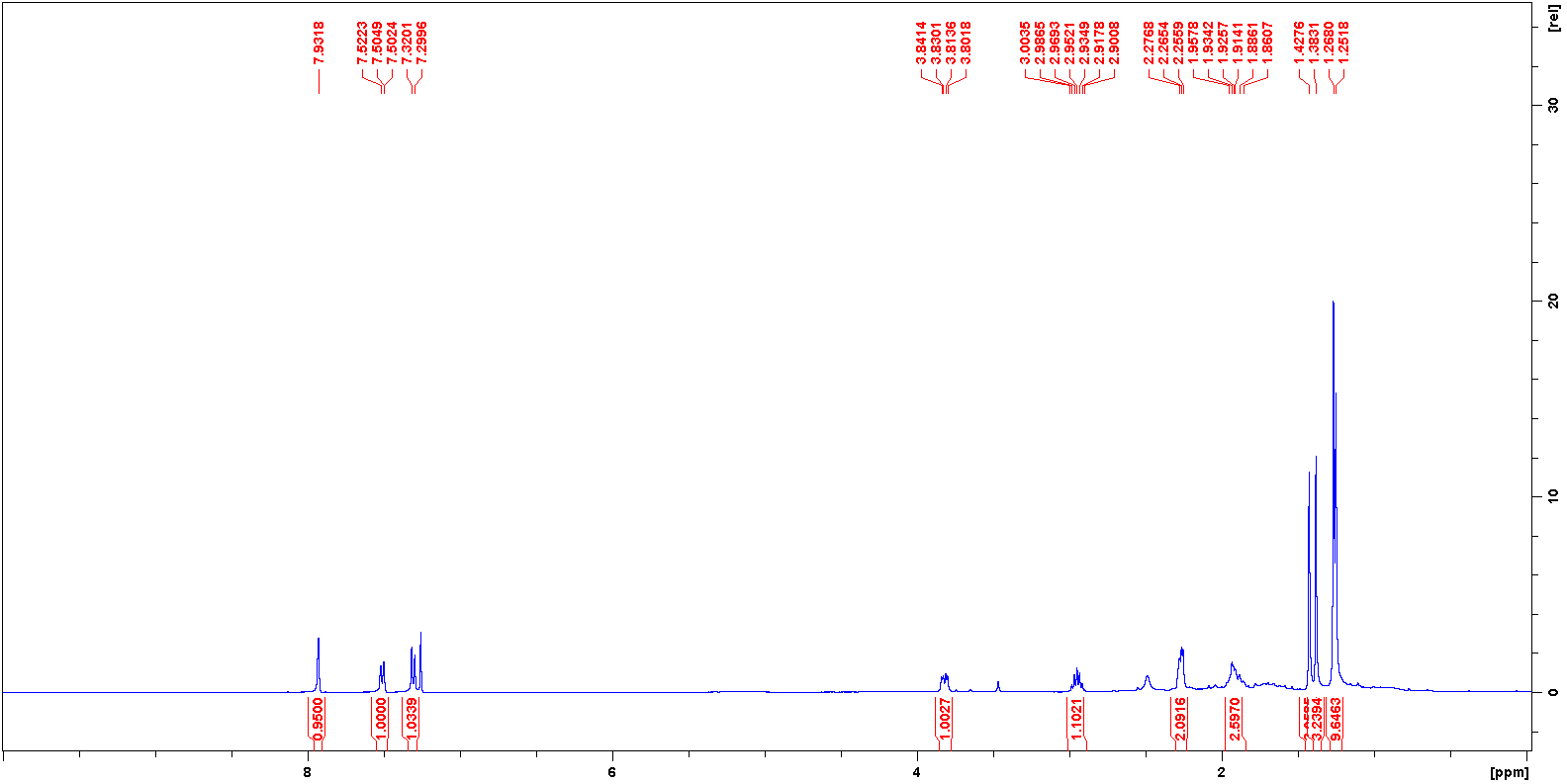


**Figure S11.** ^1^H NMR (400 MHz, CDCl_3_) spectrum for **2**


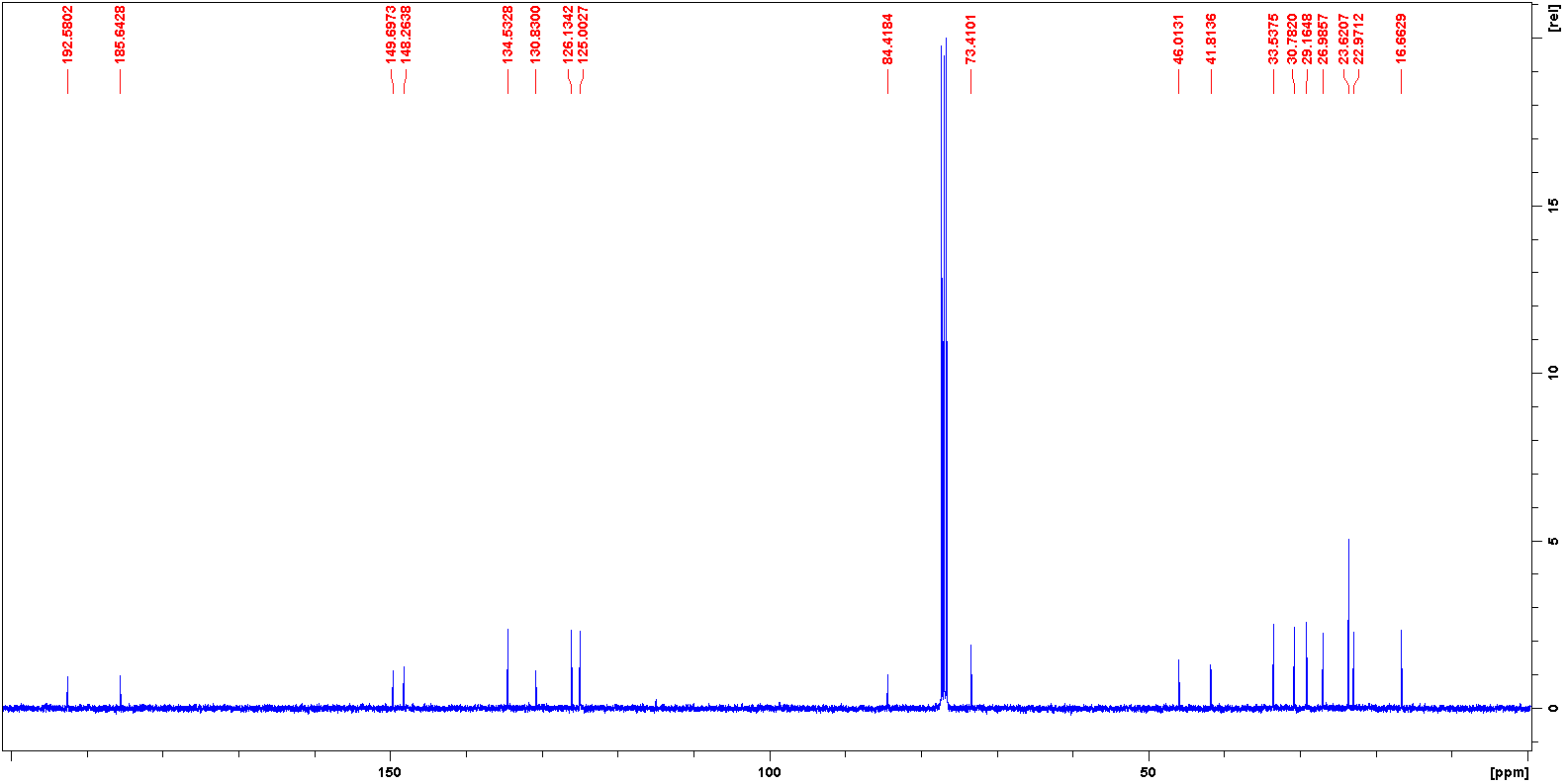


**Figure S12.** ^13^C NMR (100 MHz, CDCl_3_) spectrum for **2**


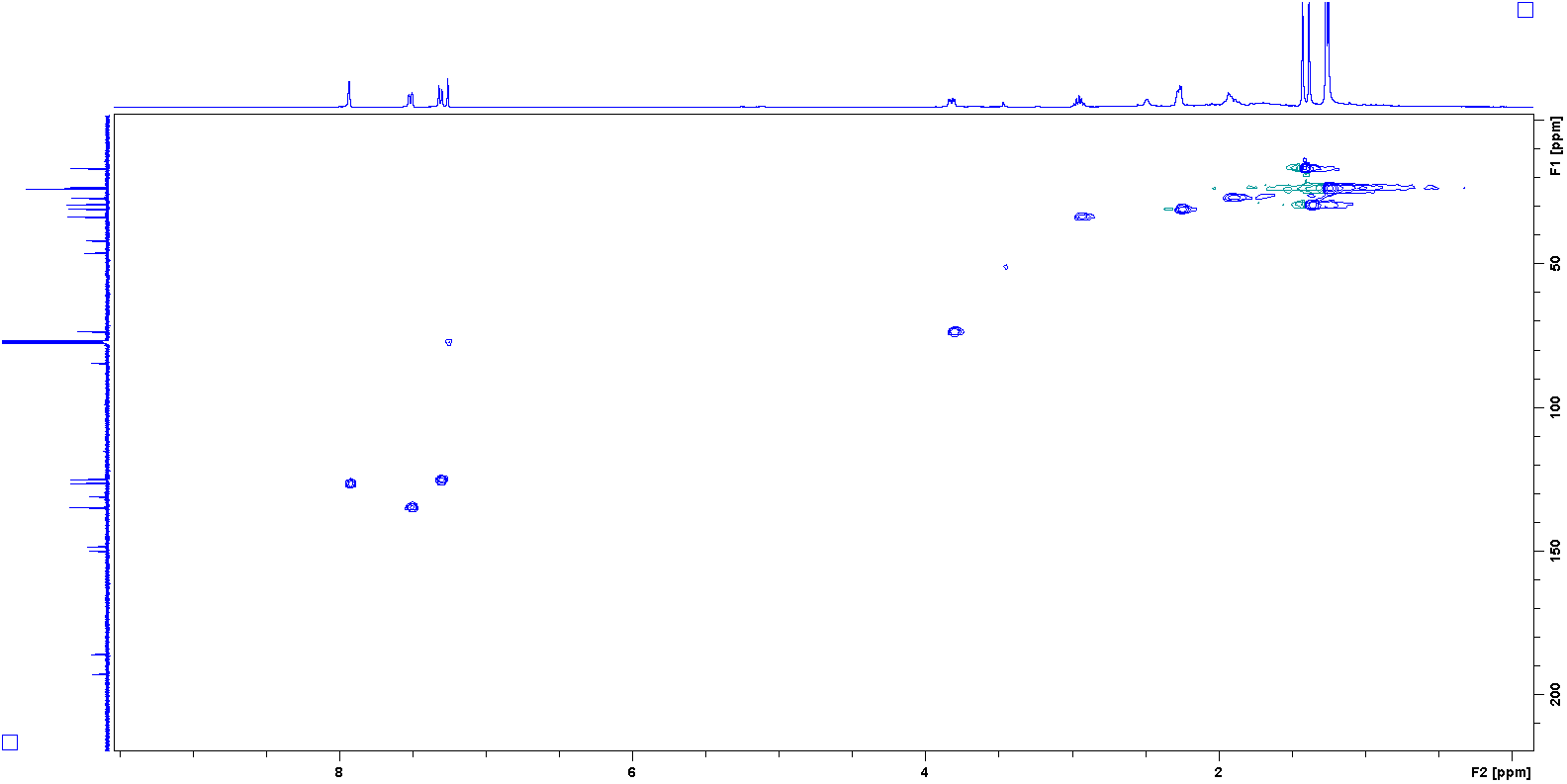


**Figure S13.** HSQC (400 MHz, CDCl_3_) spectrum for **2**


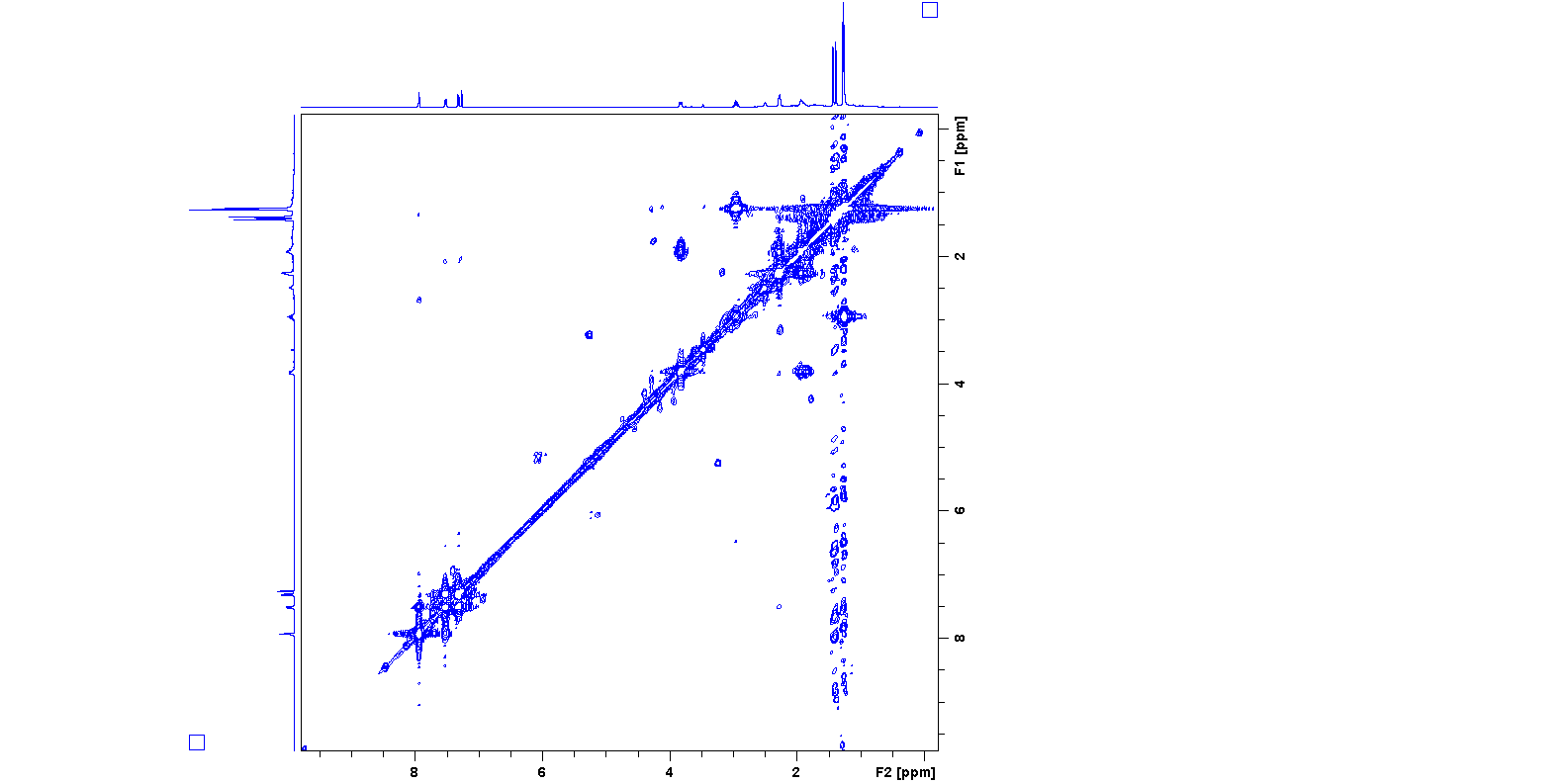


**Figure S14.** ^1^H-^1^H COSY (400 MHz, CDCl_3_) spectrum for **2**


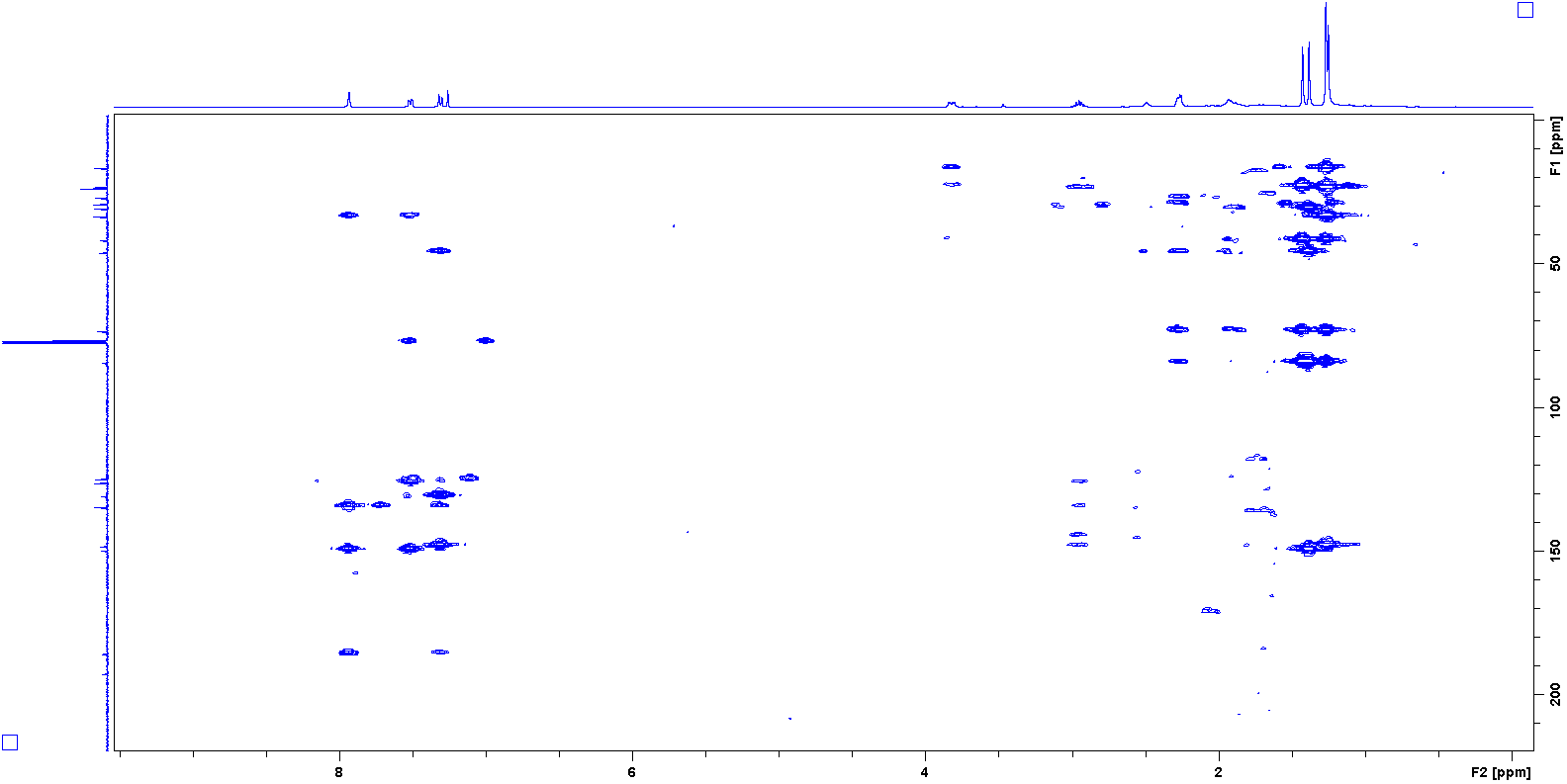


**Figure S15.** HMBC (400 MHz, CDCl_3_) spectrum for **2**


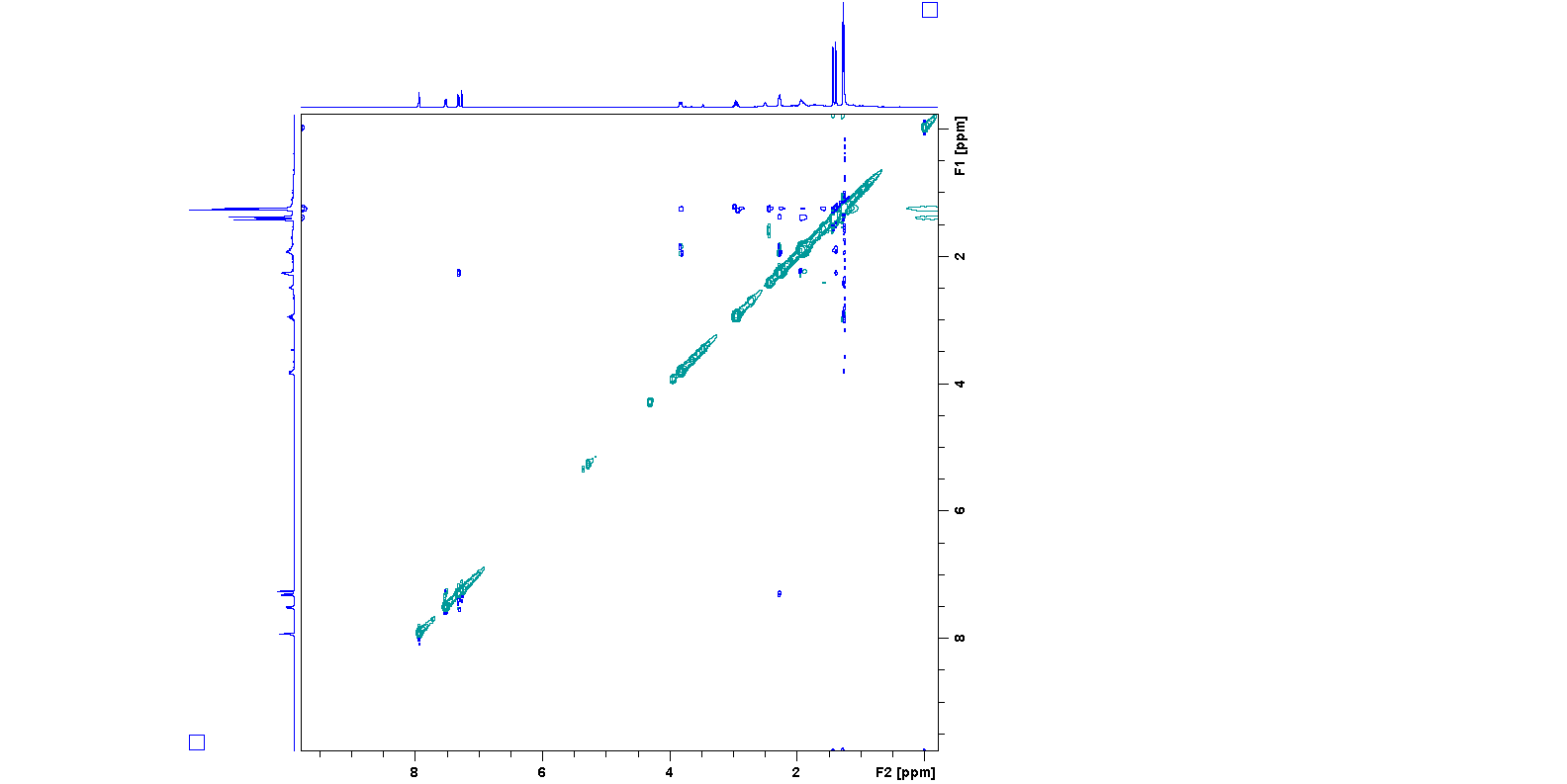


**Figure S16.** ROESY (400 MHz, CDCl_3_) spectrum for **2**

# 5. The screenshots of the checkCIF file of 1


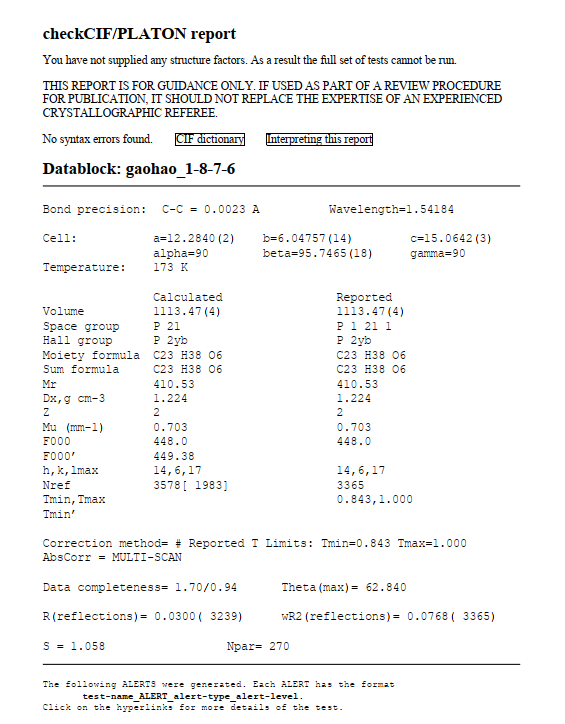


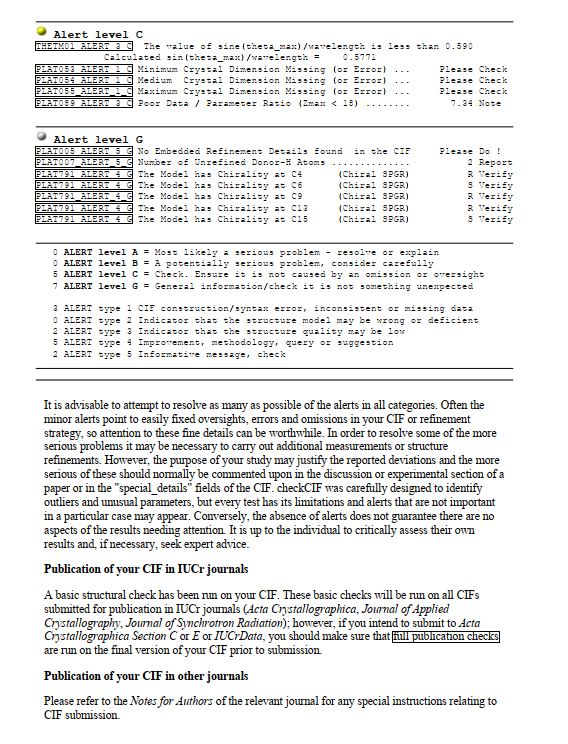


**Figure S17.** The screenshots of the checkCIF file of **1**
